# Complex karyotype including ring chromosome 11 in a patient with acute myeloid leukemia: case report

**DOI:** 10.1590/1516-3180.2016.0252150217

**Published:** 2017-08-21

**Authors:** Maria Helena Faria Ornellas, Maria Christina Paixão Maioli, Stella Beatriz Sampaio Gonçalves de Lucena, Elenice Ferreira Bastos, Tatiana Silva Chaves, Karina Vieira de Melo, Marilza de Moura Ribeiro-Carvalho, Thomas Liehr, Gilda Alves

**Affiliations:** I Associate Professor, Pathology Service, Universidade do Estado do Rio de Janeiro (UERJ), Rio de Janeiro (RJ), Brazil.; II Adjunct Professor, Hematology Service Universidade do Estado do Rio de Janeiro (UERJ), Rio de Janeiro (RJ), Brazil.; III Associate Professor, Hematology Service, Universidade do Estado do Rio de Janeiro (UERJ), and Postdoctoral Research, Research Coordination, Instituto Nacional de Câncer (INCA), Rio de Janeiro (RJ), Brazil.; IV Biologist, Hematology Department, Universidade do Estado do Rio de Janeiro (UERJ), and Supervisor, Medical Genetics Department, Instituto Fernandes Figueira (IFF), Rio de Janeiro (RJ), Brazil.; V Biologist, Hematology Service, Universidade do Estado do Rio de Janeiro (UERJ), Rio de Janeiro (RJ), Brazil.; VI Medical Sciences Master’s Student, Hematology Service, Universidade do Estado do Rio de Janeiro (UERJ), Rio de Janeiro (RJ), Brazil.; VII Biologist and Postdoctoral Researcher, Pathology Department, Universidade do Estado do Rio de Janeiro (UERJ), Rio de Janeiro (RJ), Brazil.; VIII Biologist and Head of Molecular Cytogenetics Laboratory, Jena University Hospital, Friedrich Schiller University, Institute of Human Genetics, Thüringen, Germany.; IX Biologist and Professor, Universidade do Estado do Rio de Janeiro (UERJ), and Researcher, Research Coordination, Instituto Nacional de Câncer (INCA), Rio de Janeiro (RJ), Brazil.

**Keywords:** Leukemia, myeloid, acute, Leukemia, Obesity, Chromosome aberrations, Cytogenetic analysis

## Abstract

**CONTEXT:**

Complex karyotypes in acute myeloid leukemia (AML) are characterized by an overall low response rate with frequent relapses after clinical treatment.

**CASE REPORT:**

Here, we describe the case of a 61-year-old obese female with clinically diagnosed AML who presented a complex karyotype involving an uncommon abnormality: ring chromosome 11. Immunophenotypic analysis confirmed the diagnosis. Classical and molecular cytogenetic analyses, using GTG banding and FISH (fluorescence in situ hybridization), revealed the presence of complex structural rearrangement involving r(11), add(12)(p13), der(5) and der(13).

**CONCLUSION:**

Molecular cytogenetic analysis is suitable for better identification and characterization of chromosomal rearrangements in AML. Case reports like this, as well as population-based studies, are necessary for understanding the karyotypic changes that occur in humans.

## INTRODUCTION

Acute myeloid leukemia (AML) is a heterogeneous group of diseases. In some cases, patients have satisfactory survival, whereas in others, the course has a dismal prognosis. The risk factors include obesity and chromosomal aberrations. Recent studies have suggested that obesity is a risk factor associated with AML.^1^^,^^2^^,^^3^

Classically, karyotyping has formed a powerful independent prognostic indicator in this group of diseases.^4^^,^^5^ It serves to identify biologically distinct subsets of disease and has been widely used to provide the framework for risk-adapted treatment approaches. Three subgroups can be distinguished:

1. AML with normal karyotype;
2. AML with primary balanced chromosomal aberrations; and
3. AML with unbalanced karyotype abnormalities characterized by gains and/or losses of usually larger regions of the genome and no known primary balanced abnormality.^4^

Complex karyotypes are defined as three or more independent chromosomal abnormalities in one genome. AML patients with such abnormalities are characterized by a low overall response rate, and often present relapses after clinical treatment.^5^ Ring chromosomes are considered to be a rare finding in these diseases.^6^

Here, we describe a case with a complex karyotype involving uncommon chromosomal abnormalities in an obese patient. We also present a review of the literature focusing on studies in which ring chromosome 11 was found in AML cases.

## CASE REPORT

A 61-year-old woman was registered at Pedro Ernesto University Hospital (HUPE) in December 2010, with a three-month history of dizziness, precordial pain and adynamia, a weight loss of 5.5 kg and fever. Her medical history was remarkable for obesity and hypertension. Physical examination showed pallor, rare petechiae and no palpable lymph nodes. Laboratory analysis revealed a hemoglobin concentration of 5.5 g/dl, platelet count of 25 x 10^3^/µl and leukocyte count of 19.58 x 10^3^/µl. Serological tests were negative for anti-HIV1/2, anti-HTLV1/2, anti-HBc and anti-HBS. Bone marrow immunophenotypic analyses revealed 23% blasts; positivity for CD34, HLADR and CD33; and negativity for CD7, CD19, CD10, CD117, CD36 and CD15, characterizing acute myeloid leukemia (AML), FAB classification M4.

The patient was treated with one cycle of cytarabine and daunorubicin to induce remission and four consolidation cycles of cytarabine. Hematological remission was achieved after the first cycle of cytarabine/daunorubicin (one month afterwards).

**Figure 1:**
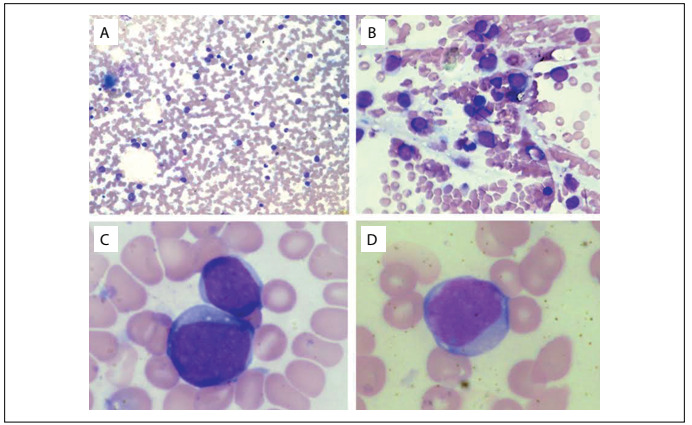
Bone marrow smears at the time of relapse of acute myeloid leukemia. A) Bone marrow presenting blastic cell infiltration, 20 X; B) Bone marrow presenting blastic cell infiltration, 40 X; C) Blast, 100 X; D) Blast, 100 X.

**Figure 2:**
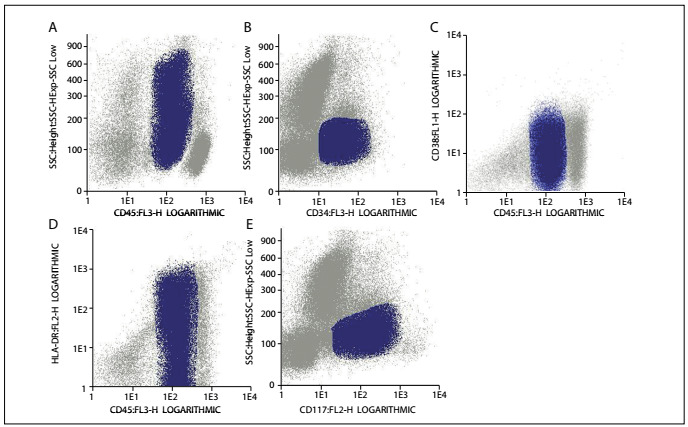
Immunophenotypic profile at the time of relapse. Dot plots revealing 71.2% of blasts positive for CD45 (A), CD34 (B), CD38 (C), HLADR (D) and (E) CD117.

**Figure 3:**
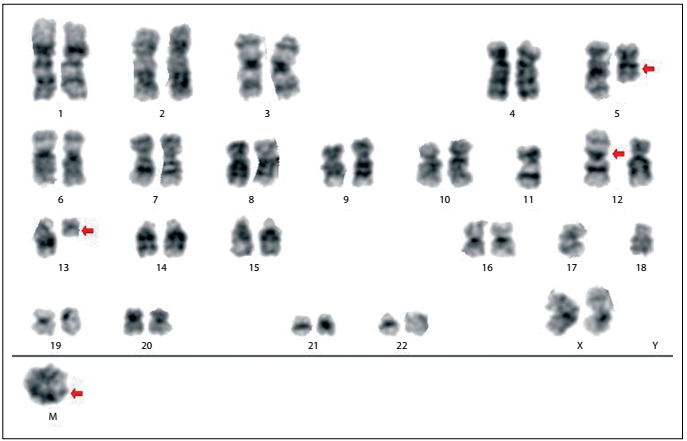
GTG banding. Complex karyotype determined through GTG banding: 46,XX, der(5), r(11), add(12)(p13) and der(13)[15].

**Figure 4:**
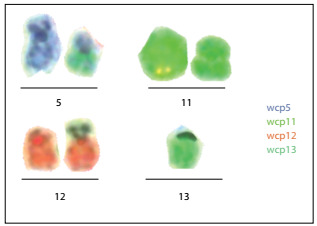
Fluorescence in situ hybridization (FISH) technique using whole chromosome painting (WCP). Chromosomes 5 (blue), 11 (green), 12 (red) and 13 (green), showing a complex translocation involving all chromosomes tested. Final karyotype: 46,XX, del(5)(q), r(11), t(11?;12;13) and der(13)t(11?;12;13).

Nevertheless, she relapsed in August 2011. At that time, a myelogram showed the presence of blasts (Figure 1) and an immunophenotypic analysis revealed that the blasts were 71.2% positive for HLADR, CD33, CD38, CD117, CD13, CD15, CD64, CD33, CD13 and CD14. The immunophenotypic profile of this case at relapse is shown in Figure 2. A molecular analysis was positive for AML-ETO/t(8;21) rearrangement and negative for PML-RARA/t(15;17) and CBFb-MyH11/inv(16)/t(16;16) rearrangements. A cytogenetic analysis using GTG banding revealed a complex karyotype of 46,XX, r(11), add(12)(p13), der(5?) and der(13?)[15] (Figure 3). For further clarification, fluorescence in situ hybridization (FISH) was performed, applying whole chromosome probes (wcp) for chromosomes 5, 11, 12 and 13 (Figure 4). The molecular cytogenetic results were as follows: 46,XX, del(5)(q), r(11), t(11?;12;13) and der(13)t(11?;12;13). A new conditioning regimen (FLAG) was started, but the patient died due to septic shock in September 2011, 293 days after diagnosis.

Ethics committee approval: CAAE #56621716.5.0000.5259.

### Review of ring chromosome 11 and AML

The literature was reviewed through the MEDLINE (PubMed) and LILACS databases (see Table 1 for details on strategy and results). For the LILACS database, the keywords in Portuguese were: “aberrações cromossômicas”, “cariótipo complexo” and “leucemia mielóide aguda”. In PubMed/MEDLINE we searched for “acute myeloid leukemia” and “chromosomic aberrations” and “ring chromosome” and “case reports”.

**Table 1:** Search of the literature in medical databases for case reports on ring chromosome 11 in association with acute myeloid leukemia

Database	Search strategies	Papers found	Related papers
MEDLINE (via PubMed)	(“ring chromosome”, “chromosomic aberrations”, “complex karyotype”, “acute myeloid leukemia” AND “leukemia, myeloid, acute”[MeSH Terms]) OR (“leukemia”[All Fields] AND “myeloid”[All Fields] AND “acute”[All Fields]) OR (“acute myeloid leukemia”[All Fields]) OR (“acute”[All Fields] AND “myeloid”[All Fields] AND “leukemia”[All Fields] AND “ring”[All Fields] AND “11”[All Fields] AND (“chromosomes”[MeSH Terms] OR “chromosomes”[All Fields] OR “chromosome”[All Fields])	66,877	22
LILACS (via BVS)	“leucemia mieloide aguda” and “leucemia” and “mielóide”, “aguda”, “aberrações cromossômicas”, “cariótipo complexo”	582	0

Articles were selected if they met the following inclusion criteria:

1. Indexed articles published between January 1, 1975, and October 20, 2016;
2. Letters to the editor, case presentations, case series, original research reports and reviews;
3. Clinical research articles on adults;
4. Articles written in the following languages were included: English, French, Portuguese and Spanish;

The exclusion criteria were:

1. Articles published outside of proposed period;
2. Other published articles not specified in the inclusion criteria;
3. Clinical research articles on children;
4. Experimental clinical research articles;
5. Articles written in languages not specified in the inclusion criteria.

Manuscripts that met the inclusion criteria were retained for full analysis. Any disagreements were resolved through further discussion involving an additional author.

The PubMed search identified 66,877 articles involving AML, of which 22 were eligible.^7^^,^^8^^,^^9^^,^^10^^,^^11^^,^^12^^,^^13^^,^^14^^,^^15^^,^^16^^,^^17^^,^^18^^,^^19^^,^^20^^,^^21^^,^^22^^,^^23^^,^^24^^,^^25^^,^^26^ The search in LILACS identified 582 articles and none of them were eligible.

Among the 22 studies selected, 34 patients presenting ring chromosome 11 were described (Table 2). Gender and age were reported in 27/34 cases, with mean ages for women and men of 62 and 60 years and medians of 61 and 63 years, respectively. The French-American-British (FAB) classification was given for 20/34 patients, and the distribution was: M0 (1 male), M1 (2 females and 2 males), M2 (2 females and 3 males and 1 gender not informed), M4 (3 females and 4 males), M5a (1 female) and M6 (1 female). Along with ring chromosome 11, all the 34 patients presented a complex karyotype.

**Table 2:** Patients’ characteristics and French-American-British (FAB) classification according the literature for acute myeloid leukemia involving ring chromosome 11

Reference	Gender/age	FAB	Outcome
Andreasson et al.^7^	F/62	Not informed	Not informed
Avet-Loiseau et al.^8^	F/53	M4	Died after diagnosis
Cigudosa et al.^9^	M/69	Not informed	Not informed
	F/59	Not informed	Not informed
Dastugue et al.^10^	M/57	Not informed	Died after 5.7 months
El-Rifai et al.^11^	F/52	M1	Still alive
Fischer et al.^12^	NI	Not informed	Not informed
Groupe Français de Cytogénétique Hématologique^13^	F/89	Not informed	Died after 1 month
Gisselsson et al.^14^	M/72	M4	Not informed
Johansson et al.^15^	M/72	M4	Not informed
Koka et al.^16^	F/61	Not informed	Died 7 days after treatment
Lindvall et al.^17^	F/74	M1	Not informed
	F/61	M4	Not informed
Liozon et al.^18^	M/65	M0	Died after 6 months
Mamuris et al.^19^	Not informed	M2	Not informed
Michaux et al.^20^	F/69	M6	Not informed
Mrózek et al.^21^	F/53	M4	Not informed
Poppe et al.^22^	M/63	M1	Not informed
	M/72	M1	Not informed
	F/46	M2	Not informed
Sárová et al.^23^	M/28	M2	Not informed
Schoch et al.^24^	NI	Not informed	Not informed
	NI	Not informed	Not informed
	NI	Not informed	Not informed
	NI	Not informed	Not informed
	NI	Not informed	Not informed
Streubel et al.^25^	M/54	M4	Not informed
Tanaka et al.^26^	M/72	M2	Not informed
Whang-Peng et al.^27^	M/45	M4	Died after 6 months
Zatkova et al.^28^	M/60	Not informed	Not informed
	M/50	Not informed	Not informed
	F/56	M5a	Not informed
	M/63	M2	Not informed
	F/76	M2	Not informed

Table showing 34 cases of acute myeloid leukemia involving ring chromosome 11.

## DISCUSSION

Although our patient satisfactorily tolerated chemotherapy and achieved complete remission after one cycle, a relapse occurred eight months later. The observed resistance to chemotherapy might be a possible explanation for treatment failure, but the expression of multidrug-resistant genes was not tested.

According to our records, obesity was the only lifestyle-related risk factor in cancer presented by our patient. She was considered to have class II/III obesity (body mass index, BMI > 35). Obesity is a chronic inflammatory condition, characterized by increased production of pro-inflammatory cytokines and adipokines, presence of hyperinsulinemia and insulin resistance and elevated levels of insulin-like growth factors.^1^^,^^2^^,^^3^^,^^29^ It has been suggested that obesity is an adverse prognostic marker in patients with cancer.^7^^,^^8^^,^^29^^,^^30^ It is well known that overweight and obesity are associated with increased incidence and mortality due to cardiovascular disease, diabetes mellitus and certain types of cancer, including leukemia. Epidemiological, case control and meta-analysis studies have correlated obesity with poor prognosis for AML.^31^^,^^32^^,^^33^^,^^34^ In addition, Finn et al. reported an association between obesity and cytogenetic categories.^35^ Several studies have considered that obesity might confer poor prognosis in different ways.^3^^,^^32^^,^^33^^,^^34^^,^^35^^,^^36^^,^^37^^,^^38^^,^^39^ For example, the mean elimination half-life of doxorubicin is longer in obese patients than in normal patients, thus increasing its toxicity to these patients. Adipocytes are also mesenchyme-derived cells and were previously considered to play only a passive “space filling” role in the bone marrow cavity. An inverse correlation between increasing numbers of adipocytes and active hematopoiesis in bone marrow is consistent with the recent identification of adipocytes as negative regulators of hematopoiesis.^29^ Other proposed mechanisms for the negative association of obesity with AML include impaired immune function due to chronic elevation of tumor necrosis factor alpha (TNFα), decreased T lymphocyte production, increased leptin and increased insulin-like growth factor activity, which are involved in hematopoiesis and survival of myeloid cells.^33^^,^^34^^,^^35^^,^^37^^,^^38^

At the time of relapse, our patient also presented a complex chromosomal karyotype involving at least four chromosomes. She was positive at the molecular level for AML-ETO [t(8;21)] rearrangement, e.g. der(12), and the marker had the capacity to carry parts of chromosomes 8 or 21.^39^^,^^40^^,^^41^ However, none of these points could be tested, because of the limitations of the material available. The AML1/ETO fusion protein is essential for development of t(8;21) AML and is well recognized for its dominant-negative effect on the coexisting wild-type protein AML1. It is associated with 12% of the cases of de novo AML and up to 40% of the cases of AML subtype M2 of the French-American-British classification. Furthermore, it has also been reported in a small portion of M0, M1 and M4 AML samples. Chromosome karyotyping and reverse transcription polymerase chain reaction (RT-PCR) results cannot be coincidental. The incidence of AML1/ETO is 5-10% higher when molecular biology approaches are used.^20,^^39^^,^^40^^,^^41^ The prognosis for AML/ETO-positive cases in the absence of t(8:21) has been reported to be poor, as was found in our case.^41^

For the chromosomal abnormalities found here, i.e. ring chromosome and translocations, it needs to be noted that ring chromosomes are considered to be rare in hematopoietic cancer (less than 10%).^6^ With regard to ring chromosome 11, this abnormality has only been found in 34 AML patients (Table 2). The outcomes of 26 of these 34 patients were not reported in the papers, but among the 8 with outcomes reported, 7 died, and only 1 was alive at the time of publication of the data. Our patient died nine months after admission. These data strongly suggest that presence of ring chromosome 11 is associated with poor prognosis in leukemia cases.

Ring chromosome 11 may carry the important leukemia-related gene MLL (mixed lineage leukemia)/KMT2A, which encodes a DNA-binding protein that methylates histone H3. MLL is a frequent target for recurrent translocations in acute leukemia cases, which can be classified as acute myeloid leukemia (AML), acute lymphoblastic leukemia (ALL), or mixed lineage (biphenotypic) leukemia (MLL). Interestingly, leukemia with translocations involving MLL shows poor prognosis. More than 50 different MLL fusion partners have been identified, and it has been observed that MLL fusion proteins lose H3K4 methyltransferase activity, thus generating transformation capacity.^42^ Unfortunately, we were unable to study the breakpoint cluster region involved in this case, and future studies will be necessary to elucidate whether ring chromosome 11 in leukemia cases carries a rearranged MLL gene and what the mechanism underlying its gene expression are.

## CONCLUSION

Molecular cytogenetic analysis is suitable for better identification and characterization of chromosomal rearrangements in acute leukemia. Single case reports, as well as population-based studies, are necessary for providing further insights into karyotypic changes that take place in human leukemogenesis.
